# Protein Biomarkers of Periodontitis in Saliva

**DOI:** 10.1155/2014/593151

**Published:** 2014-04-22

**Authors:** John J. Taylor

**Affiliations:** Institute of Cellular Medicine & Centre for Oral Health Research, School of Dental Sciences, Newcastle University, Framlington Place, Newcastle upon Tyne NE2 4BW, UK

## Abstract

Periodontitis is a chronic inflammatory condition of the tissues that surround and support the teeth and is initiated by inappropriate and excessive immune responses to bacteria in subgingival dental plaque leading to loss of the integrity of the periodontium, compromised tooth function, and eventually tooth loss. Periodontitis is an economically important disease as it is time-consuming and expensive to treat. Periodontitis has a worldwide prevalence of 5–15% and the prevalence of severe disease in western populations has increased in recent decades. Furthermore, periodontitis is more common in smokers, in obesity, in people with diabetes, and in heart disease patients although the pathogenic processes underpinning these links are, as yet, poorly understood. Diagnosis and monitoring of periodontitis rely on traditional clinical examinations which are inadequate to predict patient susceptibility, disease activity, and response to treatment. Studies of the immunopathogenesis of periodontitis and analysis of mediators in saliva have allowed the identification of many potentially useful biomarkers. Convenient measurement of these biomarkers using chairside analytical devices could form the basis for diagnostic tests which will aid the clinician and the patient in periodontitis management; this review will summarise this field and will identify the experimental, technical, and clinical issues that remain to be addressed before such tests can be implemented.

## 1. Introduction

### 1.1. The Clinical Importance of Periodontitis

Periodontitis and gingivitis are the most common forms of periodontal disease; these disorders are caused by disruption to normal homeostatic processes by numerous bacterial species found in subgingival dental plaque [1] and are modified by environmental and genetic factors [1, 2]. Gingivitis is a superficial inflammation of the gingiva (gums) and, although gingivitis is very prevalent, this disorder is effectively reversible with oral hygiene regimens. Periodontitis is a substantial destructive inflammatory condition of the anatomical structures which surround and support the teeth, namely, the gingiva, the periodontal ligament, and the alveolar bone [2]. This results in tissue injury including loss of connective tissue attachments and alveolar bone destruction. Consequently, periodontitis often results in loose teeth, pain, and impaired mastication and is a common cause of tooth loss [2]. Furthermore, periodontitis is time-consuming and expensive to treat and, therefore, prevention, early detection, and management of extent of the disease are critical issues [3]. Also, periodontitis patients have significantly poorer physical, psychological, and social oral-health-related quality of life measures as compared to periodontal healthy individuals [4].

There is a global variation in the prevalence, severity, and progression of periodontitis [2, 5]. The prevalence of periodontitis is 5–15% of adults globally [5] with some geographic variation; for example, in Asia the prevalence is as high as 15–20% [6]. Some 9% of the UK population suffer from advanced periodontitis according to the 2009 Adult Dental Health Survey (ADHS) as compared to 6% as recorded by the 1998 ADHS suggesting that there is an increasing trend toward more severe disease in the UK, possibly due to greater retention of natural teeth [7]. Also, some 15% of the UK population over 55 years of age have severe periodontal disease.

Clinical and epidemiological evidence reveals an association between chronic periodontitis and a number of systemic conditions, most notably diabetes and cardiovascular disease (CVD) [8, 9]; these associations are likely to be mediated by common pathogenic pathways [10, 11]. There is also evidence from a number of cross-sectional studies for an association between periodontitis and obesity [12] and some suggestion of an inverse relationship between sustained physical activity and periodontitis [13], although there is a recognised need for prospective cohort studies to firmly establish the clinical and pathogenic associations between these conditions [8]. Thus, an ageing population making poor diet and lifestyle choices is increasing the healthcare burden of periodontitis worldwide. Furthermore, many other diseases have periodontal manifestations including systemic infectious disease (e.g., HIV/AIDS) and some rare genetic disorders (e.g., Papillion-Lefèvre Syndrome) [2]. Significantly, the results of several meta-analyses of clinical studies show that treatment for periodontitis is associated with an improvement in glycaemic control in diabetic patients suggesting that management of periodontitis may have beneficial effects beyond oral healthcare [8]. It is established that smoking is a major risk for incidence and progression of periodontitis [14] and smoking cessation has a favourable impact on periodontitis [15]. The complex relationships of periodontitis with other diseases and risk factors suggest that the elements involved in maintenance of periodontal homeostasis, breakdown, and repair are similarly complex (see below).

### 1.2. The Role of Biomarkers in the Clinical Management of Periodontitis

Currently, there are no dependable tests to diagnose and predict progression of periodontitis. Although clinical diagnosis of periodontitis based on visual and radiographic assessment in addition to measurement of pocket depth, tissue attachment, and “bleeding on probing” (BOP) in different locations in multiple teeth is well established in dental practice, these are time-consuming procedures for the periodontist [2]. There is a requirement for tests that tell the clinician more than the conventional diagnostic tools at his/her disposal, for example, visual changes, clinical assessment (e.g., BOP), and radiographs; these traditional diagnostic procedures give an indication of severity and, therefore, reflect historical disease activity but not current disease activity and they do not identify susceptible individuals who might be at risk of future periodontitis [16]. The application of scientific evidence and patient-specific information is now considered to be central to effective clinical management of periodontitis [17]. Lack of evidence-based knowledge of individual patients' disease may lead to clinical mismanagement, for example, failure to identify disease activity and inappropriate antimicrobial therapy [3]. The requirement for reliable biomarkers to distinguish progressive periodontitis from normal biological processes is considered fundamental to identify periodontitis at an earlier or even preclinical stage, to initiate preventative pretreatment, and also to conduct epidemiological studies [3, 18].

The concept of a biomarker arose from the recognition of the appeal of being able to monitor health status, disease susceptibility, progression, resolution, and treatment outcome with respect to a number of common medical conditions [19]. Biomarkers have been defined as “cellular, biochemical, molecular, or genetic alterations by which a normal, abnormal, or simply biologic process can be recognized or monitored” [20]. Biomarkers must be indicative of physiological health, pathological processes, and/or response to therapy. Also, biomarkers must be discriminatory, robust, and validated in clinical studies. However, although a number of potential biomarkers are under investigation for diagnosis of oral and systemic diseases, a suitable marker for the detailed investigation of periodontal disease remains to be fully characterised.

### 1.3. Salivary Proteins as Biomarkers of Periodontitis

The holy grail of biomarker research in periodontology is to develop a “high impact diagnostics” which have a significant impact on clinical decision making, patient outcomes, and healthcare economics [21] equivalent to existing clinical tests, such as prostate specific antigen testing, HIV viral load analysis, HbA1c, and cholesterol monitoring. Many studies of potential biomarkers in the periodontitis literature have historically focussed on analysis of gingival crevicular fluid (GCF) which is a serum transudate originating from the gingival blood vessels which lie close to the epithelial surface of the dentogingival junction, as reviewed elsewhere [22–24]. Although a number of promising markers have been identified, analysis of GCF reflects disease activity at individual tooth sites rather than the whole mouth, a fact which complicates interpretation. There are also technical issues concerning the use of GCF: collection of GCF is a time-consuming, skilled and cumbersome procedure. Also, measurement of collected GCF volume and analyte concentrations is fraught with difficulty due to the very small volumes obtained. Nonetheless, a number of promising biomarkers identified in GCF have also been revealed to be potential salivary biomarkers for periodontitis [24].

Saliva comprises the components of exocrine secretions of the oral salivary glands as well as GCF and elements of dental plaque and the diet; the anatomy and physiology of saliva secretion and the detailed composition of saliva are reviewed elsewhere [20, 25]. It has been recognised for some years that saliva shows particular promise in terms of identifying useful biomarkers and developing convenient technologies to measure these in the clinic and (potentially) at home [19, 26]. Saliva is advantageous as it is abundant and readily accessible through noninvasive and painless procedures which will make clinical trial involvement more attractive for the patient. Also, it is cheap to collect and does not require trained medical or dental staff, and collection is not hampered by issues relating to clotting. It is possible to detect markers in saliva at low levels although some high abundant proteins in saliva, for example, amylase, albumin, and immunoglobulins, may interfere with the detection of less abundant proteins [27]. Salivary analysis has been proposed for widespread assessment of systemic disease including cancer, viral infections, and screening and investigation of oral disease [19, 28]. This includes an FDA approved at-home test for HIV based on analysis of saliva. Other applications of salivary analysis include drug detection and genetic analysis, for example, for pharmacogenetic applications.

Salivary diagnostic tests for periodontal disease include those based on analysis of molecular components, periodontal pathogens, and DNA for putative genetic risk [28, 29]. In terms of periodontal diagnosis, whole saliva not only is advantageous for its ease of collection but also has elements which reflect the activity of all periodontal sites and therefore provides an indication of disease status in the mouth as a whole rather than at individual sites as with GCF analysis. However, salivary analysis of periodontal pathogens has not proven successful as a diagnostic marker for periodontitis [30, 31]. This perhaps reflects the fact that clinical presentation of disease is not necessarily explained by the content and anatomical distribution of dental plaque and its constituent bacteria [3]. Also, our knowledge of the genetic basis for periodontitis is in its infancy so, whereas analysis of salivary DNA is possible, there are no universally accepted genetic tests applicable to periodontal disease diagnosis. For information relating to the use of genetic markers and microbiological analysis in periodontal disease diagnosis the reader is referred to excellent recent reviews [31, 32]. Other mediators which have also been investigated as candidate biomarkers for periodontitis including Ca^2+^, cortisol, hydrogen sulphide, and 8-hydroxy-deoxyguanosine have also been the subject of recent review [31]. The present review will be limited to a discussion of salivary proteins as biomarkers of periodontitis and their clinical utility.

## 2. Aetiology and Pathogenesis of Periodontitis

### 2.1. Inflammatory Processes in Periodontitis

Although the microbial components of the subgingival plaque biofilm are the aetiological factors, the pathogenesis of periodontitis is a complex interaction between the microbiota and host tissues modified by environmental factors in particular smoking, age, systemic disease, and genetic susceptibility. Our knowledge of the complexity, dynamics, and properties of the dental plaque biofilm is substantial [33]; nonetheless, a major paradigm shift in the understanding of periodontitis was the appreciation that the nature and extent of the host response to the dental plaque biofilm are a fundamental determinant in susceptibility and progression in periodontal disease [34–36]. Indeed, the relationships between different presentations of periodontal disease (e.g., gingivitis, periodontitis), disease progression, and histological features are well-established [36, 37] and, in terms of immunopathogenesis, substantial (albeit mostly indirect) evidence exists to relate immune cell diversity and distribution to the action of certain molecular mediators and, in particular, cytokines [36, 38]. More recently, we have developed an intricate knowledge of the processes that drive innate responses to plaque bacteria [1], the activation of adaptive immune responses (and especially T-cell subsets) [39], and the integration of immune regulation and connective tissue (including bone) homeostasis [40]. The molecular basis of resolution of chronic inflammation is also under scrutiny and has revealed new possibilities for therapeutic intervention [41].

**Figure 1:**
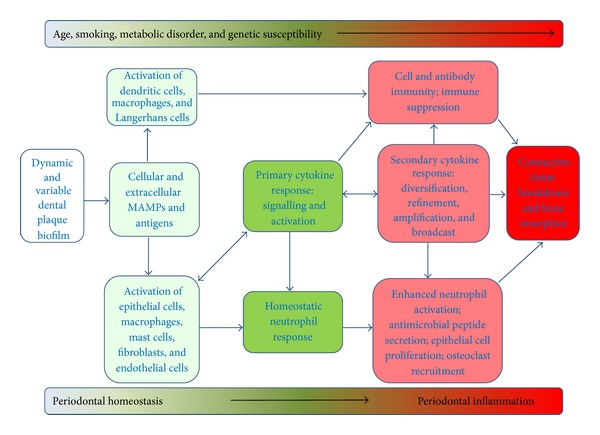
Schematic to illustrate the pathogenesis of periodontitis. The dental plaque biofilm is complex, dynamic, and variable; it is subject to quantitative and qualitative ecological shifts in response to changes in the local environment (e.g., pH changes), changes in localised immune regulation, and extrinsic factors such as smoking. Bacteria in dental plaque signal the local tissue cells and immune cells via intrinsic and secreted microbe-associated molecular patterns (MAMPs) such as lipopolysaccharide (LPS) and specific antigens (e.g., fimbrial proteins). The healthy periodontium is maintained by an effective innate response to a commensal (nonpathogenic) microflora in dental plaque which is restricted to the gingival/plaque margin and in which neutrophils play a pivotal role regulated by low levels of cytokines such as IL-1*β* and IL-8. An ecological shift in dental plaque towards a more pathogenic microflora dominated by species such as* Porphyromonas gingivalis*,* Treponema denticola*, and* Tannerella forsythia* leads to an enhanced immune response through enhanced stimulation of cytokine responses from a wide range of periodontal and immune cells. The development of periodontitis is driven by an exaggerated activation of intrinsic periodontal cells, a heightened primary and thereafter secondary cytokine response leading to activation of innate effector responses and in particular recruitment and activation of neutrophils (in response to elevated IL-1*β* and IL-8) and osteoclasts (in response to RANKL). Enhanced local activity of neutrophils in the periodontium is reflected by increased levels of MMP-8 (neutrophil collagenase), MMP-9 (neutrophil gelatinase), and *β*-glucuronidase among other enzymes. Activated macrophages and T- and B-lymphocytes may also contribute to the cytokine milieu through secretion of TNF-*α*, IL-12, IL-17, and IL-18 and the balance of these proinflammatory cytokines with immunosuppressive mediators such as IL-10 and TGF-*β* may be an important determinant of disease progression. Persistence of this proinflammatory response, coupled with aberrant resolution, leads to tissue destruction characteristic of periodontitis which involves loss of the soft connective tissues of the periodontium (lamina propria of the gingiva and the periodontal ligament) and alveolar bone which eventually leads to compromised tooth function. The presentation and progress of periodontitis are influenced by a number of secondary factors such as age, smoking, coexisting metabolic disorders (e.g., diabetes, obesity), and genetic susceptibility.

There are many molecular signals which regulate inflammatory processes such as periodontitis [42] but the present discussion will be limited to proteinaceous mediators such as cytokines and homeostatic enzymes as these are fundamental in driving immune-inflammatory responses and have been the subject of considerable study in periodontal research [36, 43]. A simplified scheme for initiation and progression of periodontitis is presented in Figure 1. The processes and interactions illustrated in Figure 1 occur naturally in healthy individuals in response to resident commensal oral microflora and this “low-grade inflammation,” although not detectable at the clinical level, is protective in nature, serving to prevent the entry of bacteria and their products into the oral tissues [1, 36]. An ecological shift in the plaque biofilm microbiota in which pathological bacterial species begin to dominate triggers enhanced immune responses which may persist leading to dysregulated and nonresolving chronic inflammation and hence periodontitis [1].

Epidemiological studies established that, in a homogenous population with even plaque distribution and oral hygiene practices, periodontal disease experience is not evenly distributed [44]. Furthermore, it is a common clinical observation that some patients do not develop periodontitis despite poor oral hygiene and others who have good oral hygiene practices (and good general health) occasionally suffer severe disease. Formal clinical studies reveal that the extent of inflammation does indeed vary among individuals for a given dental plaque challenge and has led to the assertion that secondary factors modify periodontitis susceptibility [45]. The dogma is that individuals who are susceptible to periodontitis mount an excessive, dysregulated immune response to plaque bacteria leading to tissue breakdown; individual susceptibility is determined by complex interplay between secondary factors such as genetic and epigenetic elements, age, gender, smoking, and systemic health which all influence the immune response [2, 36]. However, we have not, as yet, fully characterised quantifiable analytes which reflect these interacting factors and which allow us to identify susceptible individuals before they develop periodontitis and that is one of the clear goals of biomarker research.

### 2.2. Cytokines and Periodontitis

The biochemical pathways leading to cytokine secretion during the development of periodontitis involve signalling via the interaction of microbe-associated molecular patterns (MAMPS, e.g., LPS, DNA, fimbriae, etc.) and pattern recognition receptors (PRR, e.g., Toll-like receptors (TLR) and NOD-like receptors (NLR)) (Figure 1) which are active on a wide range of cells in periodontal tissues and infiltrating leukocytes [1, 46, 47]. Although the MAMPs are diverse, they lead to the activation of canonical intracellular signalling pathways, enhanced function of proinflammatory transcription factors such as the AP-1 and NF-*κ*B complexes with consequent effects on cell phenotype and in particular enhanced cytokine secretion (e.g., IL-8, IL-1*β*), and altered cell surface adhesion molecule expression (e.g., E-selectin, ICAMs) which in turn modify responsiveness, adhesion, and hence immune cell diapedesis [1, 46]. Further innate effector mechanisms, such as vascular changes, neutrophil chemotaxis, and function, as well as the secretion of antimicrobial peptides by neutrophils and epithelial cells, are initiated by the evolving cytokine response (Figure 1). Cytokines also activate adaptive immune responses by stimulating antigen-presenting cells (APCs), such as dendritic cells, Langerhans cells, and macrophages and regulate development of functionally diverse antigen-specific T-cell subsets once naive T-cells have been activated by APCs [36, 39]. Thus, T-cell subsets regulate antibody production by B-cells (TH_2_ cells), cell killing by T-cells and NK cells (TH_1_ cells) as well as enhancing (TH_17_ cells) or suppressing (T_reg_ cells) immune functionality [39]. Histologically, T-cells and plasma cells are prominent elements of the leukocyte infiltrate at later stages of the developing periodontal lesion [36, 38]. Immune cell activation is sustained by autocrine and paracrine loops and the evolving cytokine profile (the nature of which is dependent on the tissue milieu) serves to amplify, broadcast, diversify, and refine immune regulation (Figure 1). Persistence of pathogenic microbiota within the plaque biofilm however leads to an increasingly active proinflammatory cytokine response in the periodontium and progression of the periodontal lesion [36]. Epithelial cell proliferation in the periodontium is a prominent histological feature of periodontitis progression and serves to increase the barrier function of the epithelial tissues of the periodontium [47]. However, gingival fibroblasts are lost (by apoptosis) as the leukocyte infiltrate increases and MMPs (mostly from infiltrating neutrophils) increase collagen degradation causing connective tissue destruction including loss of the periodontal ligament (PDL) which normally serves to attach the tooth to the periodontium. In the advanced lesion, osteoclasts are recruited and activated leading to alveolar bone recession. Loss of soft (PDL and gingival connective tissue) and hard (alveolar bone) tissue leads to the loose teeth (and eventually tooth loss) periodontal tissues, characteristic of periodontitis [40].

There is substantial evidence for a key role of numerous cytokines in the initiation and regulation of immune responses in periodontitis, and, critically, in mediating tissue destruction (mainly through activation of host MMPs) [36, 46]. A consensus paper of The 7th European Workshop on Periodontal Disease recently highlighted IL-1*β*, TNF-*α*, IL-6, and receptor activator of nuclear factor kappa-B ligand (RANKL) as being the cytokines for which there is the most substantial evidence for having a central role in cytokine networks in periodontal diseases [48].

IL-1*β* is produced by a wide range of periodontal tissues and immune cells and, as such, is considered to have multiple roles in innate and adaptive immune responses to plaque bacteria which feature in the pathogenesis of periodontitis [49]. IL-1*β* acts (often in synergy with TNF-*α* and prostaglandin E_2_ (PGE_2_)) to induce many of the vascular changes associated with inflammation and in particular to regulate neutrophil emigration from the circulation into the periodontium. In adaptive immunity, IL-1*β* stimulates antigen presentation by APCs and influences T-cell development and phenotype. Studies of the expression of IL-1*β*, TNF-*α*, and PGE_2_ in oral fluids and periodontal tissues in periodontal disease endorse the important role of these mediators in pathogenesis and, critically, this is supported by the results of investigations of their effect in animal models (including key studies using cytokine antagonists) [49, 50]. Thus, IL-1*β*, TNF-*α*, and PGE_2_ will all activate osteoclast activity, MMP secretion, and alveolar bone resorption in chronic periodontitis.

IL-6 is secreted by a wide range of cells in the periodontium, probably as a secondary response to IL-1*β* and TNF-*α* activity. IL-6 is important in regulation, development, proliferation, and activity of key immune cells (B-cells, T-cells and monocytes) as well as osteoclasts (which develop from the monocyte lineage) [36, 47]. lL-6 is a powerful stimulator of fibroblast MMP secretion and is likely a key cytokine in the propagation of the inflammatory response to plaque bacteria at a number of levels [51].

Alveolar bone loss is a critical feature of disease progression in periodontitis and there is an increasing recognition of the important regulatory interactions between bone metabolism and inflammation [24, 52, 53]. RANKL stimulates bone resorption and is upregulated by IL-1*β* and IL-6 among other cytokines; thus the ratio of RANKL and its natural antagonist osteoprotegerin (OPG) is a particularly important factor in determining bone cell resorption and turnover [52, 54] and this ratio is elevated in periodontitis [24, 36].

### 2.3. Matrix Metalloproteinases and Periodontitis

There are some 23 MMPs in man which comprise a structurally related but genetically distinct superfamily of zinc-dependent endoproteases [55]. MMPs are categorised according to their perceived main substrate but it is now clear that MMPS have complex and overlapping enzymatic activities and will digest a wide variety of peptide sequences, found in a number of protein substrates and few of which are targets unique to individual MMPs [56–58]. MMPs are synthesised by the majority of cell types in the periodontium including fibroblasts, keratinocytes, endothelial cells, and osteoclasts as well as infiltrating leukocytes including neutrophils and macrophages [58]. MMPs have a wide range of fundamental physiological roles which include tissue development, homeostasis, and repair as well as roles in immune responses, including antigen processing and presentation in addition to cell migration [57]. MMPs are regulated at a number of levels including cytokine and growth factor-regulated gene transcription, processing, and activation (by other proteases in situ) [56, 57]. Inhibition of MMP activity through the formation of complexes with other molecules such as tissue inhibitors matrix-metalloproteinases (TIMPs) and serum glycoproteins such as *α*_2_ macroglobulin is another regulatory mechanism and balance between the levels of MMPs and these natural inhibitors is thought to be a crucial determinant of overall MMP activity [56, 57, 59]. In periodontal disease MMP synthesis and secretion is dysregulated, the balance of MMPs and TIMPs is altered, and the levels of neutrophil MMPs such as MMP-8 and MMP-9 are elevated, likely due to greatly increased numbers of neutrophils emigrating into the periodontitis lesion and the GCF as the result of the inflammatory process [60]. In particular, it is established that the levels of MMP-8 in GCF correlate closely with the severity of periodontal disease and that levels decline in parallel to successful periodontal therapy [16, 61, 62]. The finding that subantimicrobial disease of the doxycycline inhibits MMP-8 and can be successfully used as an adjunct therapy for periodontitis further endorses the importance of MMP-8 in periodontitis [63, 64].

### 2.4. The Spectrum of Signalling and Effector Molecules in Periodontitis

Collectively, we have much information on the role of a wide range of other host regulatory and effector molecules in the initiation, progression, and resolution of periodontitis although the sum total of information on individual mediators is limited [24, 36]. IL-17 is derived from TH_17_ cells and serves to reinforce innate responses in periodontal cells through synergising with MAMPs to enhance IL-1*β* secretion [65]. Conversely, IL-10 and transforming growth factor-*β* (TGF-*β*), for example, derived from T_reg_ cells, can modify innate and adaptive immune responses in periodontitis in a variety of ways, often, but not always, inhibiting immune responses [36, 66]. Numerous chemokines (such as CCL3 (MIP-1*α*), CXCL8 (IL-8), and CXCL10) are secreted by periodontal cells in response to primary proinflammatory mediators such as IL-1*β* and have been identified as having potential roles in periodontal pathogenesis and in particular in the recruitment and migration of neutrophils and other leukocytes to the periodontium [36, 67]. A number of growth factors are important in periodontal pathogenesis as they regulate connective tissue homeostasis and repair and can synergise with and be upregulated by proinflammatory cytokines [47]. Although there is some information on potential roles of individual growth factors such as hepatocyte growth factor (HGF), platelet-derived growth factor (PDGF), and TGF-*β* in periodontitis [68–70] given the considerable number of structural isoforms and the great functional pleiotropy of human growth factor families our knowledge of the role of these mediators is still far from complete.

The periodontium is an intricate tissue in which hard and soft tissues are uniquely juxtaposed and which comprises numerous different cell types. The periodontium is exposed to a complex, variable, and chronic bacterial challenge, in which a chronic inflammatory process is often established. Thus, it is unsurprising that the profile of molecular mediators is similarly complex, dynamic, and variable. Also, in recent years it has become apparent that although we have a much improved understanding of fundamental cellular and molecular processes relevant to periodontal pathogenesis, these are often “snapshots” of individual pathways and our understanding of how these pathways are integrated and how they map to the initiation and progression of periodontitis and, critically, the possible resolution of this disorder is limited [36]. Thus, although it is recognised that collectively these mediators constitute a complex system which has the functional properties of a network rather than existing as many parallel, linear pathways, we have yet to understand how these function in immune responses and immune-mediated disease. A property of complex networks of interacting molecular elements is that changes in the levels of individual elements do not necessarily have predictable effects on the functionality of the network and that there may be new, emergent properties not obvious from the study of individual molecules in isolation [71]. Therefore, one of the major barriers to our exploitation of this knowledge (e.g., in the identification of key biomarkers) is gaining a holistic understanding of the relative roles of the vast array of known mediators in the pathogenesis of periodontitis and currently, in terms of biomarker identification, we still rely on information from “candidate” mediator studies and the literature is dominated by many studies on a limited number of these candidate markers such as IL-1*β*, OPG, and MMP-8.

## 3. Protein Biomarkers of Periodontitis: Candidate Protein Studies

### 3.1. Cross-Sectional Clinical Studies

**Figure 2:**
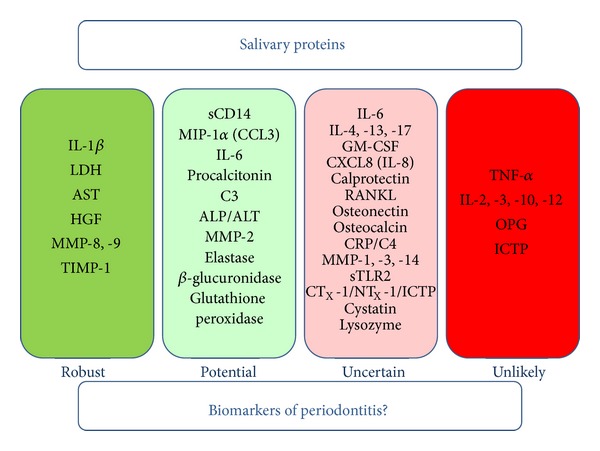
The possible role of salivary proteins as biomarkers of periodontitis. “Robust” biomarkers are defined as those salivary proteins which have been shown to discriminate between periodontitis and oral health in at least 3 cross-sectional studies (with comparatively little or no published evidence to the contrary) and for which there may be supporting evidence from longitudinal studies investigating the natural course of periodontitis and/or the effects of treatment on biomarker levels. “Potential” biomarkers are identified using identical criteria to “robust” biomarkers with the exception that there are 2 replicated cross-sectional studies showing disease discrimination in addition to possible supporting evidence from longitudinal studies but for which there may be limited contradictory studies. It is accepted that the entries in the “robust” and “potential” categories may be interchangeable depending on the existence of further studies which remain unpublished for commercial reasons. “Uncertain” biomarkers are proteins for which there are only single studies showing discrimination of periodontitis or for which there are several studies from which the evidence is contradictory. “Unlikely” biomarkers are those proteins for which there are 3 or more studies which fail to provide evidence for an association with periodontitis in the absence of any evidence to the contrary. For a more detailed description of the published research studies and the putative role of these proteins in periodontitis see the main body of the text.

Clinical studies of GCF and saliva have identified a number of promising individual mediators which are associated with periodontal disease and correlated with clinical measurements of periodontitis, and whose levels change in parallel with the clinical course of the disease and in response to treatment [24, 29, 72]. The majority of studies have been “cross-sectional” in design and have simply compared mean levels of mediators in groups of periodontitis patients with those found in healthy volunteers. These studies are thus attempting to identify “association” with periodontitis and therefore whether or not the potential biomarker fulfills the requirement of being “discriminatory.” Also, there are substantial confirmatory studies for only a limited number of these candidate biomarkers; therefore few such biomarkers can be described as “robust.” Salivary mediators analysed in these cross-sectional studies and an assessment of the possible role of these proteins as biomarkers for periodontitis are presented in Figure 2.

Evidence from studies of GCF suggests that cytokines likely form a substantial and measurable element of oral fluids [24]. There is a well-established association between elevated levels of IL-1*β* in GCF and periodontitis [24, 49]. There is strong evidence to suggest that salivary IL-1*β* is a good biomarker of periodontitis in as much as measurement of IL-1*β* can discriminate periodontitis samples from those provided by healthy volunteers [73–79] but this finding has not been replicated in all studies [80–82]. Also, one study of some 98 periodontitis patients revealed a positive correlation between levels of salivary IL-1*β* and the extent of alveolar bone loss [83].

Studies of salivary TNF-*α* levels have failed to provide evidence of association with periodontitis often because the levels of TNF-*α* in saliva are very low or nonexistent [75, 76, 78, 79, 81, 82, 84]; although one study did report significantly elevated levels of TNF-*α* in periodontitis patients as compared to healthy controls, the actual levels of TNF-*α* were very low (<4.3 pg/mL) [85].

The majority of studies of salivary IL-6 demonstrate no association with periodontitis [75, 78, 81, 82]. In contrast, there are 3 reports of significantly elevated salivary IL-6 levels in periodontitis [79, 86, 87]. However, analysis of salivary GM-CSF, IL-2, IL-3, IL-4, IL-5, IL-10, IL-12, and IFN-*γ* revealed no significant associations with periodontitis [81, 82, 88] although there is a single report of elevated salivary IL-4 and significantly lower salivary IL-17 in periodontitis [87]. Interestingly, IFN*α* levels have been found significantly elevated in the healthy individuals as compared to patients with periodontitis [79].

Although there is a paucity of data on salivary chemokines in periodontitis, 2 studies have indicated that salivary CCL3 (MIP-1*α*) levels are significantly associated with periodontitis [89, 90]. There is, as yet, no evidence that CXCL8 (IL-8) has a potential as a biomarker for periodontitis [78, 81].

Interestingly, there is evidence from 2 independent studies that levels of salivary soluble CD14 (sCD14) are elevated in periodontitis [87, 91]; sCD14 mediates the action of LPS and may promote bacterial invasion of host cells in periodontitis [92, 93]. Also, although salivary sTLR2 augments IL-8 (CXCL8) production in monocytes [94], there is evidence for diminished levels of sTLR2 in periodontitis saliva [87].

There is an emerging interest in a number of cytokines such as osteocalcin, RANKL, and OPG which are regulators of bone cell activity and, as such, mediate bone loss characteristic of periodontitis [24, 95]. However, the results of analysis for potential associations of salivary levels of these cytokines and periodontitis have been variable. Thus, one study failed to find significant differences between RANKL levels in periodontitis as compared to controls [85] although a more recent report presented data to the contrary, showing elevated levels of RANKL in saliva from periodontitis patient [96]. Also, significantly lower levels of OPG in periodontitis as compared to controls have been reported [82, 96] but other studies found no evidence for such a correlation [73, 86, 90]. The data for association of both salivary osteonectin and osteocalcin (both of which have a role in bone metabolism [97, 98]) with periodontitis are similarly inconsistent [99–103].

A number of studies of salivary biomarkers for periodontal disease have used candidate molecules identified through their established use as systemic markers of inflammatory disease in general medicine. For example, calprotectin is a neutrophil protein which is considered to be a marker of inflammation [104] and is increased in the saliva of periodontitis patients [82] as is procalcitonin, which is also a marker of systemic inflammation [105, 106]. Similarly, although 2 studies from the same group suggest that reduced levels of the acute phase protein C-reactive protein (CRP) in saliva are associated with periodontitis [84, 107], data from other studies are not consistent with this finding [80, 108]. Studies of salivary levels of the complement components C3 and C4 have revealed an association of periodontitis with lower levels of C3 found in saliva as compared to healthy controls [84, 107]. Lactate dehydrogenase (LDH), alkaline phosphatase (ALP), aspartate aminotransferase (AST), and alanine aminotransferase (ALT) are markers of cellular damage and inflammation; there is evidence for an association between salivary levels of all these mediators and periodontitis [109–114] with the exception of one study which reported no association with ALP, AST, or ALT [111] and another which failed to reproduce significant association with salivary LDH [75].

*β*-Glucuronidase is an enzyme found in neutrophil lysosomes where it has a role in the digestion of proteoglycans. *β*-Glucuronidase levels in saliva are a marker of neutrophil influx into GCF and salivary levels of this enzyme correlate with the severity of periodontitis [72, 115]. Also, measurements of salivary glutathione peroxidase, as a marker of neutrophil antioxidant capacity, show that the levels of this enzyme are significantly elevated in periodontitis [102, 116].

Saliva contains abundant antibacterial proteins such as lysozyme, and cystatins, but there is only a single report of significantly reduced levels of salivary cystatins in periodontitis [117] and the results from studies of salivary lysozyme have been contradictory [78, 117, 118].

Growth factors have diverse functions which overlap and contribute to immune responses but although elevated levels of growth factors including TGF-*β*, epidermal growth factor (EGF), and vascular endothelial growth factor (VEGF) have been reported in the GCF of patients with periodontitis [95], there is a dearth of information about salivary growth factors and periodontitis. Exceptionally, 3 independent studies demonstrated significant association of salivary HGF with periodontitis [119–122] which is consistent with similar data analysing GCF [123]. In terms of function, HGF is known to be involved in dental development [124] but, significantly, HGF is secreted by gingival fibroblasts and HGF secretion is regulated by cytokines and bacterial products; it has been hypothesised that HGF may mediate epithelial apical migration in periodontitis [124, 125].

Data from analysis of MMP in saliva in periodontitis are consistent with the findings from studies of GCF [24]. Nonetheless, studies of GCF are more extensive and there is a requirement, given the advantages of salivary analysis, to replicate these studies using saliva collection. In particular numerous more longitudinal studies have been performed and analysis of GCF levels of other members of the MMP superfamily (e.g., MMP-7, MMP-25) has identified a wider range of candidate biomarkers [24]. That neutrophil collagenase (MMP-8) activity in saliva is elevated in periodontitis patients as compared to healthy volunteers and it correlates with clinical measures of periodontitis is well-established by the results of numerous studies [73, 76, 78, 79, 82, 86, 102, 126–134]. Significantly, total collagenase activity in health is in the form of the inactive procollagenase as compared to a preponderance of activated collagenase in periodontitis saliva [128]. Furthermore, with the exception of a single study [130], TIMP-1 concentration has been found to be higher in saliva from healthy individuals as compared to periodontitis patients [128, 134, 135]. However, there is no evidence for an association of salivary MMP-1 (fibroblast collagenase), MMP-3 (stromelysin-1), or MMP-14 (a membrane-type MMP) with periodontitis [130, 134]. A key question is whether or not a particular biomarker for periodontitis cannot only identify the presence of periodontal inflammation but also whether or not it is able to determine the extent of inflammation; that is can the marker distinguish periodontitis from gingivitis? In this regard, MMP-8 seems to be a better biomarker of periodontitis than other markers such as IL-1*β* [78].

Salivary gelatinases (MMP-2 and MMP-9) and elastase have been found to be significantly elevated in periodontitis patients as compared to controls [82, 108, 129, 135, 136] although there are reports of no differences between disease and control samples with respect to salivary MMP-9 [130] and elastase [75].

Collagen matrix degradation by proteases such as MMPs leads to the release of fragments of collagen into the circulation; these peptides can be assayed as a measure of bone resorption in periodontitis [95]. One such peptide is C-telopeptide pyridinoline cross-links of type-1 collagen (ICTP), but although ICTP is elevated in GCF from periodontitis patients [95] and has been found to be elevated in periodontitis and associated with clinical measures of periodontitis [103, 134, 137], several other independent studies have failed to detect salivary ICTP in saliva from the majority of periodontitis patients [82, 83, 85, 90]. Nevertheless, other peptide markers of bone loss and osteoclast activity including C-terminal peptide of type 1 collagen (CT_X_-1), N-terminal peptide of type 1 collagen (NT_X_-1), and tartrate-resistant acid phosphatase serum type 5b (TRACP 5b) correlate with clinical periodontitis and may have promise as salivary biomarkers although these markers are not found universally in saliva samples [102, 138].

Aggressive (acute) periodontitis is a form of periodontal disease associated with rapid loss of alveolar bone and gingival attachment and as such has a distinct presentation and clinical course to the more common chronic periodontitis described above [139]. It is plausible that appropriate biomarkers may be employed to distinguish between these related disorders. For example, antibodies to* Aggregatibacter actinomycetemcomitans* (a periodontal pathogen often associated with aggressive periodontitis) and* Porphyromonas gingivalis* are thought to have a key role in protective immunity in aggressive periodontitis [140] but although these antibodies are indeed found in saliva their presence is not specific to aggressive periodontitis [141, 142]. A very limited number of studies (of comparatively few patients) have directly compared levels of mediators in aggressive periodontitis and chronic periodontitis [91, 127, 143]; with the exception of one study which reported elevated cathepsin C activity in the saliva of aggressive periodontitis patients [143] none of these studies have identified biomarkers which might be potentially specific for aggressive periodontitis. Although there is a real need for larger studies, establishing specific protein biomarkers may be challenging as there is no evidence for significant differences in the immunopathogenesis of these two clinical manifestations of periodontitis [139].

### 3.2. Longitudinal Clinical Studies

Although many markers are discriminatory, that is, exhibit significant alterations in periodontitis and robust, that is, have been found to be discriminatory in more than 3 independent studies, their clinical utility remains uncertain. Many studies outlined above have quantitatively correlated mediator levels with clinical measurements of periodontitis (e.g., bleeding on probing, clinical attachment loss, and pocket depth) although this data has not always been presented. However, it is unclear whether or not these measurements reflect current disease activity or are the result of historical disease progression or whether or not the levels of many candidate biomarkers significantly associated with periodontitis in cross-sectional studies accurately reflect the likely path of disease progression and response to treatment. Although a number of longitudinal studies have recorded changes in GCF levels of MMPs in parallel with the clinical course of periodontitis, the data for salivary analysis is rather limited and is the result of studies using clinical protocols which are very variable making meaningful comparisons between studies difficult to achieve. Therefore, the need for more substantial clinical studies very much still exists [24].

In broad terms, published longitudinal studies of salivary mediators in periodontitis may be categorised as studies of levels during the natural progression of the periodontitis in patients without therapeutic or healthcare interventions and studies of the effects of particular active healthcare regimens. In a prospective cohort study, 219 unselected subjects were monitored over a 4-year period, during which time they did not undergo any dental treatment and changes in periodontal pocket probing depth (as a measure of periodontitis) as compared to levels of salivary biomarkers and lifestyle factors; a multiple logistic model revealed that disease progression related to smoking habit and not the levels of any of some 9 protein biomarkers (IL-1*β*, MMP-8, MMP-9, lactoferrin, IgA, albumin, AST, LDH, and ALP) [144]. Significantly, levels of salivary lactoferrin, AST, and LDH were reduced in smokers, a finding consistent with the known immunosuppressive effect of cigarette smoke [144, 145]. In a longitudinal case-control study salivary biomarkers including osteonectin, HGF, IL-1*β*, IFN-*γ*, TNF-*α*, IL-4, IL-6, IL-8, and ICTP were measured in 40 subjects who exhibited significant alveolar bone loss over a 5-year follow-up period as well as in 40 age-matched subjects with no bone loss [120]. The results demonstrated a negative association between the extent of alveolar bone loss over the 5-year period and levels of salivary osteonectin and a positive association of bone loss with both IL-1*β* and HGF suggesting that measurement of these mediators at baseline may have a predictive value in monitoring periodontitis [120]. Another prospective cohort study found that levels of salivary MIP-1*α* (CCL3) and IL-1*β* (among 19 other mediators that were assayed) were both significantly elevated in 7 subjects with localised aggressive periodontitis as compared to 41 individuals who stayed healthy [89]. Moreover, the levels of MIP-1*α* in saliva were elevated prior to radiographic detection of bone loss suggesting that this mediator may be predictive of longitudinal disease progression in periodontitis and analysis of covariance suggested that salivary MIP-1*α* had the strongest correlation with periodontal destruction [89]. Although both salivary MIP-1*α* and IL-1*β* were elevated in subjects with periodontitis in this study, the level of MIP-1*α* is considered to be a more sensitive biomarker for periodontitis as levels of salivary MIP-1*α* were increased 50-fold in contrast to the mere 5-fold elevation of IL-1*β* [89]. Also, IL-1*β* is regarded as a marker of inflammation as well as tissue destruction and may not therefore be directly predictive of disease progression in periodontitis [89, 146]. There is, therefore, some evidence that certain biomarkers reflect disease progression in untreated patients but smoking habit clearly influences biomarker status [144] and, unfortunately, smoking data has not been alluded to in all studies [89, 120].

Numerous more studies have investigated the effects of various treatment regimens, including nonsurgical and surgical approaches, on salivary biomarkers in periodontitis. However, the treatment regimens applied have differed considerably between studies and, unfortunately, there are insufficient data to compare the effects of particular periodontal therapies on individual biomarkers. Nonetheless, a common finding is a decline in biomarker level in parallel with improvement in clinical measures of periodontitis. Thus, a significant reduction for active collagenase after surgical and nonsurgical treatment in both localised juvenile periodontitis and adult periodontitis patients has been recorded [60, 127]. Comparison of the behaviour of salivary proteases (not including collagenase) and glycosidases in patients treated for periodontitis revealed that although the levels of both types of enzymes declined as a result of treatment, they did not do so simultaneously [147]. Two smaller studies of the effect of periodontal treatment on salivary antibacterial proteins have yielded inconsistent data [148, 149]. There is, as yet, no evidence that salivary glutathione peroxidase is altered by treatment in periodontal disease [150]. However, systemic markers of inflammation (AST, ALT, and LDH) do decline after nonsurgical treatment for periodontitis, at least over a 4-week period [151]. A modest study of 20 periodontitis patients and 20 controls demonstrated that IL-4 decreased significantly and sTLR2 increased significantly following such treatment [87]. In a study of 39 postmenopausal women, salivary osteocalcin was shown to be inversely related to decrease in clinical measures of periodontitis [99]. Salivary MMP-8 (but not MMP-9) levels were found to be reduced after nonsurgical treatment for periodontitis in 33 patients but, interestingly, not if adjunctive doxycycline treatment was included [152]. Conversely, TIMP-1 increased after nonsurgical treatment with adjunct doxycycline treatment but not after nonsurgical treatment alone [152].

A key recent study reported the longitudinal clinical investigation of periodontitis in parallel to measurement of a panel of potential biomarkers, a cohort of 100 volunteers [153]. Significantly, the study consisted of a 6-month monitoring phase, during which no treatment was provided. During the monitoring phase there were no changes in clinical periodontitis in the volunteers and the levels of biomarkers as compared to baseline did not change significantly. This was followed by provision of appropriate treatment, followed by a 6-month “disease-recovery” phase during which disease recovery and progression were monitored [153]. It was found that the levels of MMP-8, MMP-9, OPG, and IL-1*β* declined significantly after treatment in volunteers with moderate to severe periodontitis. In a case-controlled study of some 68 patients with periodontitis, biomarkers were serially analysed throughout the course of 28 weeks when 33 patients received oral hygiene instruction (OHI) only and the remaining 35 patients had nonsurgical treatment in addition to OHI [146]. Periodontitis improved in both groups in parallel with a significant reduction in salivary MMP-8 levels. Detailed analyses revealed that levels of salivary OPG and MIP-1*α*, in addition to MMP-8, were able to discriminate patients who responded to treatment from those who did not. However, IL-1*β* and MMP-8 levels decreased significantly only in the group receiving nonsurgical treatment in addition to OHI (as compared to OHI alone); this treatment group also exhibited the greatest reduction in clinical measures of periodontal disease, suggesting that these markers most accurately reflected clinical changes in periodontitis [146]. Receiver-operator characteristic analysis showed that MMP-8 was the best biomarker to reflect response to therapy [146].

Given the complexity of the disease pathogenesis and progression and the recognised interindividual differences in disease experiences it is likely that multiple biomarkers will be required in order to provide full diagnostic efficacy [142, 153]. The concept of “biomarker signatures” in which multiple rather than individual markers provide more robust association with periodontitis has been endorsed by the work of Ebersole et al. [79]; in a study of 30 healthy adults and 50 patients with periodontitis, salivary IL-1*β*, IL-6, and MMP-8 were all significantly elevated in the patient group and receiver-operator characteristic analysis indicated that all 3 mediators had sensitivity and specificity values in the range 80–97% and positive predictive values of >90% [79]. These authors have previously described substantial intraindividual “random” variation in the salivary levels of these mediators in healthy individuals [154] but, despite this, simultaneous analysis of a combination of analytes (IL-1*β*, IL-6, and MMP-8) clearly has a high capacity to distinguish periodontitis patients from healthy individuals [79].

It is possible that different tests will be applicable to screening for disease susceptibility as compared to those required for monitoring of therapy and treatment selection [21]. Interestingly the power to predict differences between periodontal disease categories (moderate to severe periodontitis as compared to mild periodontitis and gingivitis) was increased when measures of salivary MMP-8 were combined with those of MMP-9, OPG, and calprotectin and quantification of periodontal pathogens such as* P. gingivalis* and* Treponema denticola* in dental plaque [82]. Also, a decline in the levels of MMP-8, MMP-9, OPG, and IL-1*β* occurred in parallel with changes in the levels of several periodontal pathogens in dental plaque [153]. Using hierarchical clustering analysis the authors found that the combined levels of biomarkers (both molecular and bacterial) successfully predicted progression of periodontitis and disease stability during the recovery phase for the majority of cases. Parallel analysis of mediators in serum samples suggested that this approach was not sensitive to the clinical course of periodontitis in agreement with previous findings [155, 156]. This approach supports the concept of a “biological signature” of multiple distinct analytes in saliva encompassing several different elements of host-pathogen interactions in periodontitis acting as biomarkers [79, 82, 153].

## 4. Protein Biomarkers of Periodontitis: Proteomic Studies

The majority of studies characterising salivary protein biomarkers in periodontitis published thus far have been based on markers derived from pre-existing information on the molecular pathogenesis of periodontitis; there needs to be a more open, unbiased approach to biomarker identification in periodontitis. Thus, high throughput proteomics holds promise for disease-associated biomarker identification [157] and substantial progress has been made in the proteomic analysis of saliva through a combination of sophisticated approaches to protein separation and advances in mass spectrometry technology [157, 158]. The salivary proteome has now been identified; it contains some 1166 proteins [159] and proteomic studies confirm the commonality of the salivary proteome and plasma proteins [159, 160]. Proteomic approaches have already identified salivary biomarkers of both Sjogren's syndrome and oral cancer [19, 161, 162].

A number of preliminary studies investigating the proteomic profile of saliva in periodontitis using a combination of 2D electrophoresis and mass spectrometry have been published [163–165]. Although this approach determines the salivary protein profile in an unbiased analysis, the approach is only sensitive enough to detect proteins with a relatively high abundance and many mediators, for example, cytokines and MMPs, are not in sufficiently high concentrations to be detected [164, 166]. Thus, in a comparative study of salivary proteins from 5 patients with generalised aggressive periodontitis (GAgP) and 5 healthy patients, some 11 proteins were found to be altered in the GAgP patients; these included some high abundance proteins already known to be associated with inflammation including *α*-amylase, lactoferrin, IgG2, IgA2, and albumin [163]. Increased vitamin-D binding protein was also found to be associated with localized aggressive periodontitis (LAgp) for the first time [163]. Similar changes in *α*-amylase, Ig heavy chain, and albumin (along with decreased cystatin levels) in saliva from patients with chronic periodontitis and gingivitis have also been noted in independent proteomic studies [165, 167]. Other proteins exhibited decreased expression in GAgP: these included elongation factor 2, 14-3-3 sigma, lactotransferrin, and short palate, lung, and nasal epithelium carcinoma associated protein 2 (PLUNC2) [163]. PLUNC2 (also known as parotid secretory protein) was also found to be decreased in the saliva of patients with active periodontitis as compared to saliva from the same patients after treatment [164]. Furthermore, three independent proteomic studies have confirmed the presence of increased levels in periodontitis saliva of two of the S100 family of calcium binding proteins which have known roles in regulation of inflammation [163, 166, 168]. A recent study has applied a highly sensitive liquid chromatography/mass spectrometry protocol (which obviates the need for 2D gel electrophoresis) to the analysis of the salivary proteome in 20 patients with periodontitis as compared to saliva from 20 healthy volunteers [166]. This study identified some 20 proteins which were differentially expressed in periodontitis saliva and most, including MMP-8, MMP-9, *α*_2_-macroglobulin, and complement C3, have previously been identified as potential biomarkers in conventional analysis of periodontitis saliva [166]. The authors note that, in agreement with other studies using mass spectrometry analysis of fractionated salivary proteins, only small differences between the levels of these proteins distinguish periodontitis saliva from saliva from healthy volunteers [166]. Gene ontology and pathways analysis for the data from this study reveal that the differentially expressed mediators are mostly associated with the acute phase responses and regulation of inflammation [166]. Although proteomic analyses of this kind provide the unbiased detection of salivary proteins, issues of sensitivity, recovery, and processivity remain a hindrance to achieving a comprehensive global analysis of salivary proteins; however, new technological developments are addressing these issues and there is the promise that this approach will deliver information to enhance candidate protein studies [169].

Developments in bioinformatics will play a key role in the interrogation of data from proteomic studies, integration with other global analyses (e.g., transcriptomics and metabolomics), exploitation of knowledge of complex biochemical and cellular interactions, and ultimately, therefore, in the identification of salivary biomarkers not obvious from studies of candidate proteins [170]. A key advance in this area is the development of the salivomics knowledge database (SKB) and the salivary proteome knowledge base which is part of the human salivary proteome project [170, 171]. Salivomics is an emerging discipline which describes the study of related sets of biological molecules including the transcriptome, the proteome, and the metabolome in saliva and which, it is envisaged, will drive the development of personalised diagnostic approaches in the dental clinic [170, 171]. The SKB contains a data management resource sourcing salivomics research data which interfaces with other relevant databases [170, 171].

## 5. Future Challenges in Biomarker Identification

Biomarkers must be straightforward to analyse; the readout should be readily interpretable, and give enhanced information such as being able to predict onset, measure activity, and monitor disease progression (e.g., from gingivitis to periodontitis or in terms of severity of periodontitis or relapse after treatment) and there is real promise that a number of the markers described in the previous section may fulfill these criteria. However, there are a number of scientific, clinical, and technological challenges to achieving the successful clinical application of salivary diagnostics in the management of periodontitis [171].

### 5.1. Clinical Study Design

Despite our growing knowledge of the molecular mediators of periodontitis and the advent of salivomics, there remains a lack of substantial, well-controlled, clinical studies aimed at characterising the efficacy of individual biomarkers or combinations in monitoring periodontitis. In particular, the majority of such studies published thus far have been conducted on a cross-sectional basis rather than longitudinal design and another weakness of many studies is that they are statistically underpowered and focused on individual mediators [24, 36]. Also, there is a dearth of studies which have substantially analysed the relationship of salivary biomarkers with the natural progression of periodontal disease, for example, in long-term epidemiological studies or during an experimental gingivitis model. It is important to note that there are a number of practical issues which complicate the design and interpretation of such studies. For example, widespread adoption of uniform definitions of periodontal disease remains an issue there is subjectivity and variability of definitions employed and a consensus is required for the design of multicentre trials, for comparison of different trials, and for the correct conduct of community and epidemiological studies [172, 173]. Also, it has long been recognised that the progression of periodontitis is highly variable. Initially, it was thought that the periodontal lesion progressed in a linear fashion albeit at a rate variable between individuals [174]. Latterly, the “burst” or “episodic” hypothesis has emerged to explain the variable phases of tissue breakdown and repair around individual tooth sites in periodontitis patients [175]. It is now considered that these theories of periodontal progression are not mutually exclusive and that they represent manifestations of the cyclical destructive inflammation and improvement which occur longitudinally in individual sites [176]. Nonetheless, the progress of periodontitis and response to treatment remains complex and highly variable and therefore difficult to predict.

A major issue in clinical studies of periodontal disease is cigarette smoking which modifies numerous salivary mediators [144, 145] and strongly influences periodontitis [14, 177]. Therefore, the inclusion of smokers confounds the conclusion of many studies. Other confounding factors which ideally should be eliminated from such studies include age, gender, race, and comorbid conditions [146]. A rubric to integrate the many quantifiable elements which contribute to periodontal pathogenesis with the complex clinical course and the changes in biomarkers needs to be developed using bioinformatics and a systems biology approach; this may facilitate the provision of more detailed clinically useful information which will inform a more individualised and effective clinical management approach [46, 170, 178, 179].

### 5.2. Salivary Collection Protocols

In addition to a broadening of the biochemical analysis for biomarker into the realm of salivomics and more appropriate clinical validation studies for salivary biomarkers outlined above, there are a number of practical issues that need to be addressed before this approach is translated into a bona fide diagnostic test.

In particular, there needs to be a reliable method of salivary collection; to date, a great variety of approaches have been used which will impact on the effectiveness of the test [20, 180]. Variation in salivary flow rates, stimulatory factors, and gingival inflammation contribute to a variable salivary composition [28]. The mode of collection is also critical; some studies report the use of stimulated or unstimulated saliva and the collection of whole saliva or saliva secreted from individual glands. Also many studies do not record the time of collection, a relevant factor as many salivary components exhibit circadian rhythm [20]. Some studies have adopted an “oral rinse” protocol which is considered to be a fluid containing GCF from all the periodontal pockets and thus has the advantage, like whole saliva, of reflecting disease activity in the whole mouth rather than individual sites as with GCF collection [181], whereas whole saliva is collected by a variety of methods and its content may be affected by the flow rate.

### 5.3. Biomarker Analysis Technology

The method of analysis needs to be fit for the purpose. Although most clinical studies of salivary protein biomarkers have employed conventional ELISAs, there has been some interest in developing novel point-of-care (POC) devices which may be used in the dental clinic and eventually domestically. The real promise of salivary analysis use is the ability of the patient or clinician to directly and continuously assess disease status, progression, and therapeutic efficacy. Sensitive analysis may even allow presymptomatic diagnosis. Thus, microfluidic and microelectromechanical systems (MEMS) and nanoelectrofluidic systems (NEMS) for salivary diagnostics have been under development for at least the last decade [19]. Multiplex analysis is envisioned, perhaps involving simultaneous detection of different molecular structures such a proteins and nucleic acids [19]. Microfluidics have the advantage of using low sample and reagent volumes; microelectronics facilitate the development of miniaturised chairside and handheld devices suitable for use in the dental clinical and home in the absence of specialised laboratory facilities. In combination, they promise to provide simultaneous and rapid measurement of multiple biomarkers coupled with data storage and transmission. Any novel technology must also have commercial attractiveness [21]. Technological developments aim to optimise biomarker measurement with respect to high sensitivity, high specificity, miniaturisation, high throughput, automation, portability, low cost, high functionality, measuring multiple disease markers, and simplicity of use. POC devices may also potentially enhance epidemiological research in remote and impoverished areas which lack access to laboratory technology and appropriate sample storage and transport facilities. There are several examples of POC devices at prototype stage [80, 132, 182]; these devices potentially have greater sensitivity and linearity than conventional immunoassays. The performance of these devices will be likely dependent on reagents employed and the lack of appropriate antibodies may limit development of some biomarker assays. For example, some monoclonal antibodies to MMP-8 preferentially detect active MMP-8 whereas others may detect total MMP-8 including the biologically inactive pro-MMP-8 [181, 183]. Differences in immunoreactivity of antibodies employed in immunoassays may account for discrepancies in the results of some of the aforementioned clinical studies. However, the status of this field remains uncertain as clearly dissemination of detailed information is likely limited by commercial sensitivities and intellectual property restrictions.

### 5.4. Clinical Adoption

There needs to be an understanding of the issues relating to acceptance of these novel diagnostic approaches by dental clinicians and their patients alike. The derivation of the so-called “value propositions” is likely to be a useful approach whereby key questions such as feasibility, diagnostic accuracy, impact on patient management and outcomes, and also impact on society (including return on investment issues) are addressed [184]. Patient self-care in periodontitis is critical to the economically viable and widespread prevention of severe disease [185]; identification of biomarkers and development of simple monitoring devices may facilitate this.

## 6. Concluding Remarks

We now have substantial information suggesting that a limited number of protein biomarkers may be efficacious in diagnosis and management of periodontitis. These markers (e.g., MMP-8 and IL-1*β*) have been identified on the basis of their known role in disease pathogenesis; there needs to be an approach to the unbiased selection of markers using “salavomics” coupled with more substantial, longitudinal, clinical studies in order to develop this field further. There is also a need to develop a practical approach to chairside analysis which will enable the dental clinician to efficiently and accurately assess periodontitis disease activity. Development of effective salivary protein biomarkers for periodontitis will ultimately require a multidisciplinary approach involving biological scientists, biotechnologists, engineers, and clinicians. If these aims are realised then this will greatly enhance not only the clinical care of periodontitis patients but also our ability to conduct more effective clinical and epidemiological studies of this common, complex disease. The integration of epidemiological studies of human health and disease with detailed biomarker analysis would help to determine marker sensitivity, specificity, and applicability; the UK Biobank [21] and National Health and Nutrition Examination Survey (NHANES) in the USA [186] are examples of resources that might be thus exploited.

There has been some considerable interest in promoting the dental clinic as a venue for general medical diagnosis through physiological measurements (such as weight, height, and blood pressure), finger stick blood tests, and salivary diagnostics [21]. For example, screening in the dental clinic for patients at risk of cardiac disorders is medically beneficial [186]. Research in this area has shown that not only are dental patients willing to donate saliva for diagnostic purposes but also dentists themselves are willing to collect such samples [187, 188]. Development of a practical framework for clinical management of periodontitis using salivary biomarkers will provide a paradigm for exploring the application of general medical diagnostic procedures using saliva in the dental clinic.
